# Morphological abnormalities in *Hyalomma dromedarii* and *Hyalomma rufipes* (Acari: Ixodidae) collected from dromedary camels (*Camelus dromedarius*) in Aswan, Egypt

**DOI:** 10.1007/s10493-022-00747-2

**Published:** 2022-10-29

**Authors:** Mohammed Okely, Deon K. Bakkes, Lidia Chitimia-Dobler

**Affiliations:** 1 Entomology Department, Faculty of Science, Ain Shams University, Abbassia, Cairo, 11566 Egypt; 2 Gertrud Theiler Tick Museum, Agricultural Research Council – Onderstepoort Veterinary Research, Pretoria, 0110 South Africa; 3 Bundeswehr Institute of Microbiology, Neuherbergstrasse 11, Munich, Germany

**Keywords:** Abnormalities, Gynandromorphism, *Hyalomma dromedarii*, *Hyalomma rufipes*, Ixodidae

## Abstract

The present study reports anomalies in *Hyalomma dromedarii* and *Hyalomma rufipes* adults collected from dromedary camels (*Camelus dromedarius*) in Aswan, Egypt, between January and June 2022. A total of 52 adult ticks displayed one or several local and/or general anomalies. A wide variety of local anomalies was observed including atrophy of one or multiple legs, ectromely, absence of accessory adanal and subanal plates, fusion of adanal and accessory plates, and presence of sclerotized formation in the ventral plate, as well as abnormalities of the spiracle, anal groove, festoon, parma, and posteromedian groove. General anomalies comprised of asymmetries and gynandromorphism. Local anomalies were documented among *H. dromedarii* and *H. rufipes*, whereas general anomalies were documented only from *H. dromedarii*. The present work represents the first report of deuterogynander intrigue gynandromorphism in *H. dromedarii*, as well as the first report of morphological abnormalities in *H. dromedarii* and *H*. *rufipes* from Egypt.

## Introduction

Morphological abnormalities in ticks have not been commonly reported, and the first case of anomalies in hard ticks was reported in 1899 (Neumann 1899). This phenomenon may be caused in nature by several extrinsic or intrinsic factors including host resistance to tick infestation, high temperature and humidity, environmental pollution, or somatic and germinal mutations (Latif et al. 1988; Dergousoff and Chilton 2007; Buczek et al. 2013, 2019; Kar et al. 2015; Keskin et al. 2016; Shuaib et al. 2020). Additionally, exposure to acaricides and chemical agents may play a role too (Oliver and Delfin 1967; Buczek 2000). It is also possible that some of these factors may interact with gene regulatory networks during development to disrupt normal morphogenesis (Kittelmann et al. 2018); however, this requires further study.

Tick morphological abnormalities have been classified into general and local anomalies (Campana-Rouget 1959a, b). Gigantism, nanism, idiosomal constriction, duplication, gynandromorphism, and asymmetries are examples of general abnormalities, whereas ectromely, atrophy of one or multiple legs, asymmetry of spiracles, and abnormality in festoons and ventral plates are among local anomalies (Campana-Rouget 1959a, b; Guglielmone et al. 1999; Kar et al. 2015; Shuaib et al. 2020; Laatamna et al. 2021). Gynandromorphism is a unique type of abnormality where morphological sex characteristics may be combined in a single specimen (Martini et al. 1999; Keskin et al. 2012). Additionally, gynandromorphic abnormalities can occur simultaneously with the other abnormalities mentioned above. There are five forms of gynandromorphism in ticks according to the classification of Campana-Rouget (1959a): bipartite protogynander, where the external features of both sexes are equally represented; deuterogynander, where features of one sex are decreased to a quadrant; metagynander, where features of one sex are decreased to a small segment; gynander intriqué, a protogynander or deuterogynander in which some features of one sex are embedded in areas of the opposite sex; and mosaic gynandromorphism, where there is no definitive line separating the male from the female. The bipartite protogynander is the most widely reported among these five forms (Labruna et al. 2002; Keskin et al. 2012).

Morphological abnormalities have been described in different genera of ixodid ticks including *Amblyomma*, *Dermacentor*, *Haemaphysalis*, *Hyalomma*, *Ixodes*, and *Rhipicephalus* (Campana-Rouget 1959a; Guglielmone et al. 1999; Zharkov et al. 2000; Alekseev et al. 2007; Kar et al. 2015; Keskin et al. 2016; Chitimia-Dobler et al. 2017; Wang et al. 2019). In the genus *Hyalomma*, anomalies have been reported in *Hyalomma marginatum*, *Hyalomma aegyptium*, *Hyalomma scupense*, *Hyalomma impeltatum*, *Hyalomma excavatum*, and *H. dromedarii* (Keskin et al. 2012, 2016; Nowak-Chmura 2012; Kar et al. 2015; Shuaib et al. 2020).

Numerous studies have reported abnormalities in several tick species in Europe, Asia, and America (Keskin et al. 2016; Larson and Paskewitz 2016; Ren et al. 2016; Chitimia-Dobler et al. 2017; Molaei and Little 2018, 2020; Chong et al. 2020; Molaei et al. 2020; Diyes and Rajakaruna 2021). In Africa, documentation of morphological abnormalities in ticks remains limited and has been reported only in South Africa, Uganda, Sudan, and Algeria (Gothe 1967; Balinandi et al. 2019; Shuaib et al. 2020; Laatamna et al. 2021). In this study, local and general morphological abnormalities were investigated among hard ticks collected from dromedary camels (*Camelus dromedarius*), for the first time from Egypt.

## Materials and methods

Ticks were collected monthly from January to June 2022 from dromedary camels (n = 50) in a camel market in Aswan, Egypt (24.408642 N, 32.941018E). All tick specimens were removed from various parts of each camel’s body using fine forceps and then stored in vials containing 70% alcohol and 20% glycerol to be transported for morphological identification to the Acarology Laboratory, Department of Entomology, Ain Shams University. The collected specimens were identified based on morphological characters using identification keys (Hoogstraal 1956; Walker et al. 2003; Apanaskevich and Horak 2008; Apanaskevich et al. 2008; Okely et al. 2021). Ticks were identified and examined using a CZM4 Stereo Microscope (Labomed, Fremont, CA, USA) with an Am Scope LED-144 W-ZK white adjustable luminance and photographed using an attached MU1000 10MP microscopic camera (AmScope, Irvine, CA, USA). After the identification, ticks were deposited for future research in the Okely’s Tick Collection (Department of Entomology, Ain Shams University, Cairo, Egypt).

## Results

In total, 1248 adult ticks were collected and morphologically identified and assigned to three genera (*Amblyomma*, *Hyalomma*, *Rhipicephalus*) and four species (Table 1). Of these, morphological abnormalities were observed in 52 (4.2%) specimens, only in *H. dromedarii* and *H. rufipes* species (Table 2). No abnormalities were noted in *Amblyomma lepidum* and *Rhipicephalus pulchellus*.

**Table 1:** Hard ticks collected from dromedary camels from January to June 2022 in Aswan, Egypt

Species collected	Sex	Number of ticks collected
*Hyalomma dromedarii*	Male	1007
	Female	218
*Hyalomma rufipes*	Male	8
*Amblyomma lepidum*	Male	13
*Rhipicephalus pulchellus*	Male	2
Total		1248

**Table 2:** Numbers of detected morphological abnormalities in *Hyalomma* tick species collected from January to June 2022 in Aswan, Egypt

Type of abnormalities		Tick species	n	Rate of abnormal ticks (%)	Rate of examined ticks (%)
*General*	Gynandromorphism	*H. dromedarii*	1	1.9	0.08
	Asymmetries	*H. dromedarii* (♂)	2	3.8	0.16
*Local*	Atrophy of one or multiple legs	*H. dromedarii* (♂)	2	3.8	0.16
		*H. rufipes* (♂)	1	1.9	0.08
	Ectromely of leg	*H. dromedarii* (♂)	1	1.9	0.08
	Absence of accessory adanal plate	*H. rufipes* (♂)	1	1.9	0.08
	Absence of subanal plates	*H. dromedarii* (♂)	2	3.8	0.16
		*H. rufipes* (♂)	1	1.9	0.08
	Fusion of adanal and accessory adanal plates	*H. dromedarii* (♂)	1	1.9	0.08
	Presence of sclerotized formation in ventral plate	*H. dromedarii* (♂)	1	1.9	0.08
	Subanal plate anomalies	*H. dromedarii* (♂)	20	38.5	1.6
	Spiracle anomalies	*H. dromedarii* (♂)	3	5.8	0.24
		*H. dromedarii* (♀)	1	1.9	0.08
	Adanal plate anomalies	*H. dromedarii* (♂)	4	7.7	0.32
		*H. rufipes* (♂)	1	1.9	0.08
	Anal groove anomaly	*H. dromedarii* (♂)	1	1.9	0.08
	Festoon anomalies	*H. dromedarii* (♂)	4	7.7	0.32
	Parma anomalies	*H. dromedarii* (♂)	3	5.8	0.24
	Postermedian groove anomalies	*H. dromedarii* (♂)	2	3.8	0.16
Total			52	100	4.16

n = number of anomalous ticks

## Local anomalies

Local morphological abnormalities in *H. dromedarii* were represented by atrophy of one or multiple legs, ectromely, absence of subanal plates, fusion of adanal and accessory adanal plates, presence of sclerotized formation in ventral plate, anomalies of subanal plates and adanal plates, spiracles, anal groove, festoons, and postermedian grooves, whereas local anomalies in *H. rufipes* were represented by atrophy of one leg, absence of accessory adanal plate and subanal plate, and an anomaly of adanal plate (Table 2).

**Fig. 1:**
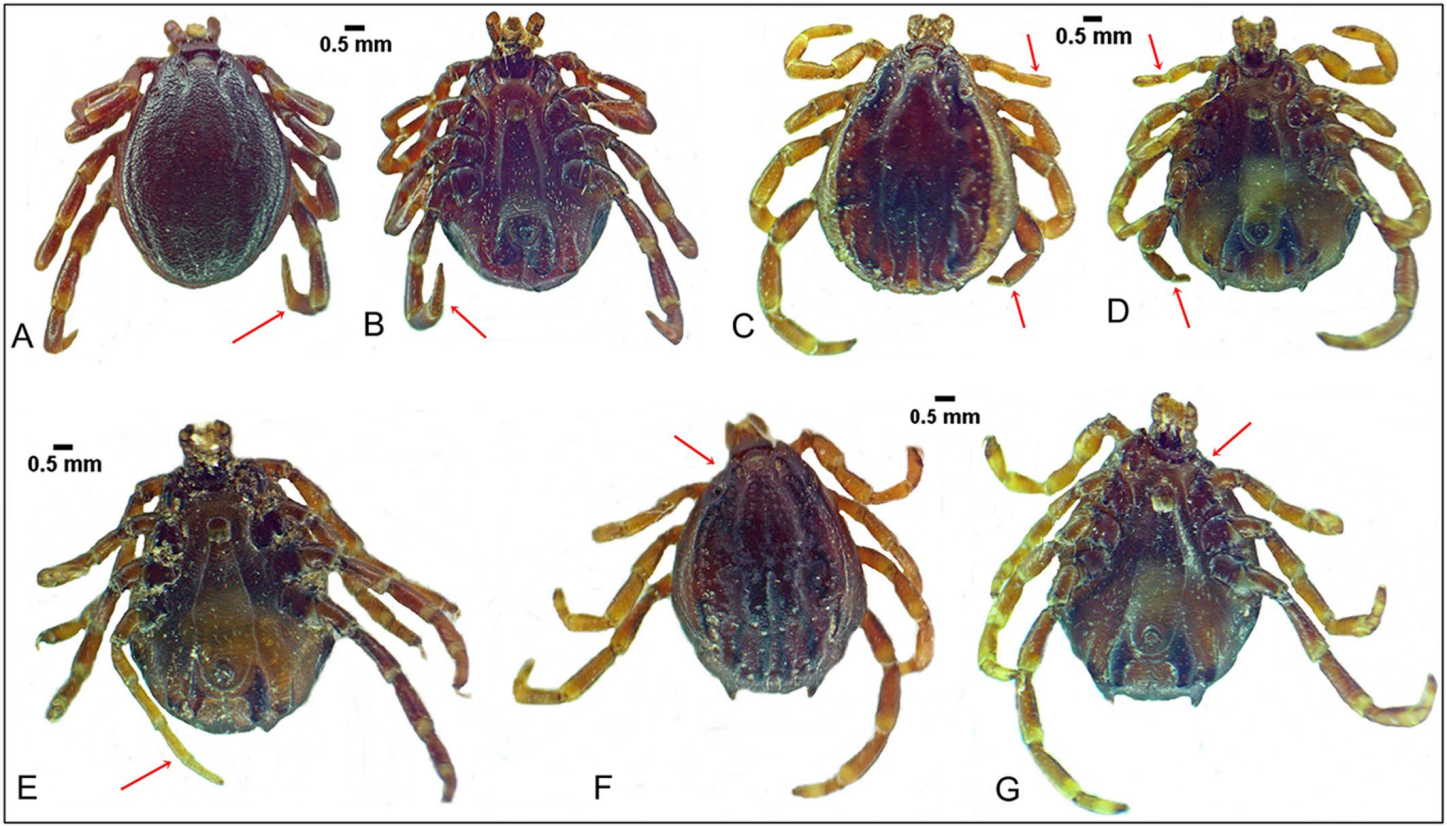
Atrophy of leg in *Hyalomma rufipes* male, dorsal view (**A**), ventral view (**B**); atrophy of legs in *H. dromedarii* male, dorsal view (**C**), ventral view (**D**, **E**); ectromely in *H. dromedarii* male, dorsal view (**F**), ventral view (**G**)

Atrophy of legs was found in three male specimens (0.24%) of the total collected ticks. One *H. rufipes* male showed atrophy of the fourth right leg associated with idiosoma deformation on the same side (Fig. 1A, B). For the two *H. dromedarii* males, one showed atrophy of all legs on the right side, which was comparatively shorter than those on the left side (Fig. 1C, D), and the second specimen had atrophy of the fourth right leg (Fig. 1E). Ectromely of the first left leg of a *H. dromedarii* male was present and associated with deformation of the anterior part of the body on the same side (Fig. 1F, G).

**Fig. 2:**
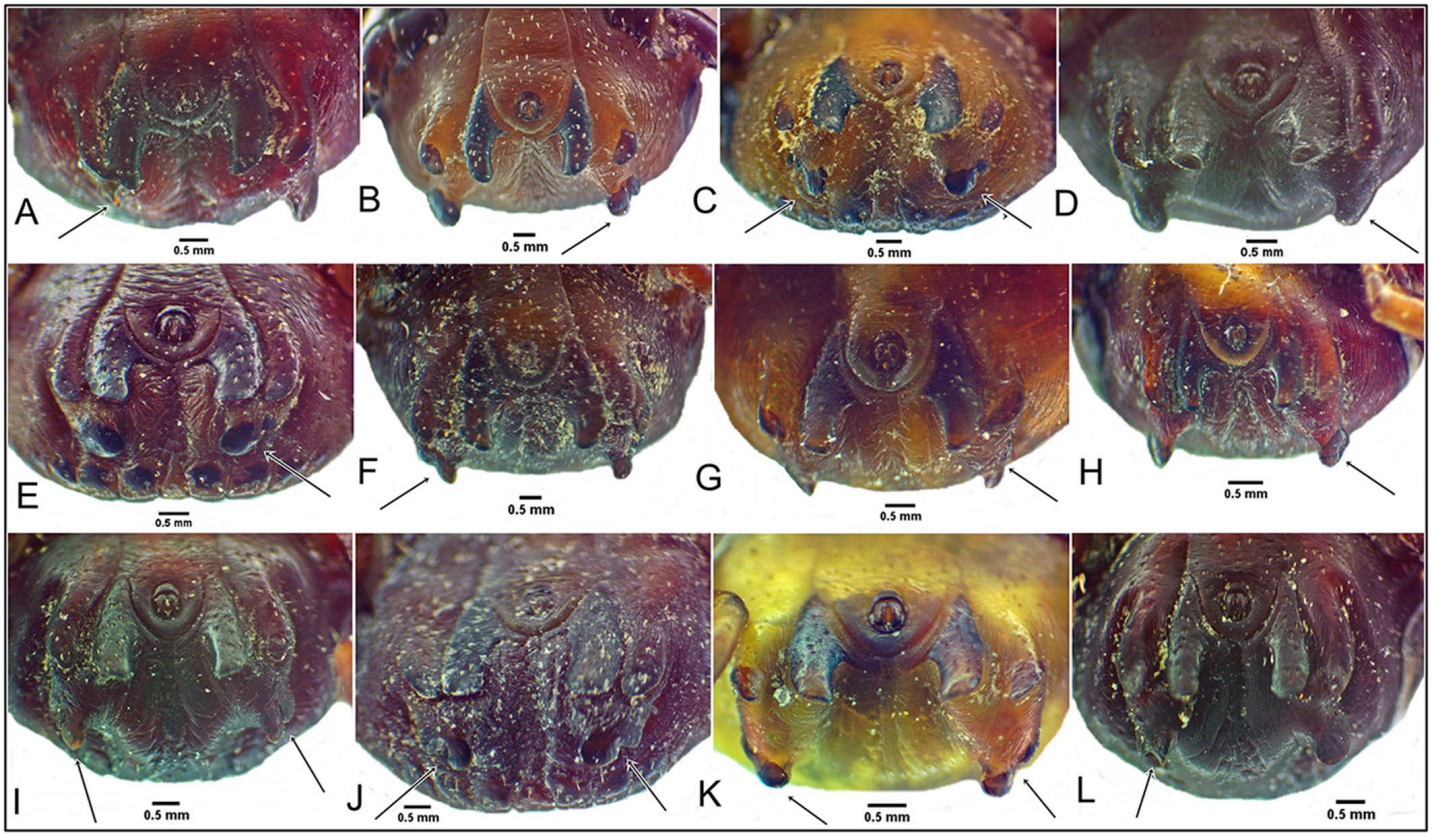
Subanal plate anomalies in *Hyalomma dromedarii* male, ventral view (**A**–**L**); fusion of adanal and accessory adanal plate in *H. dromedarii* male, ventral view (**A**)

**Fig. 3:**
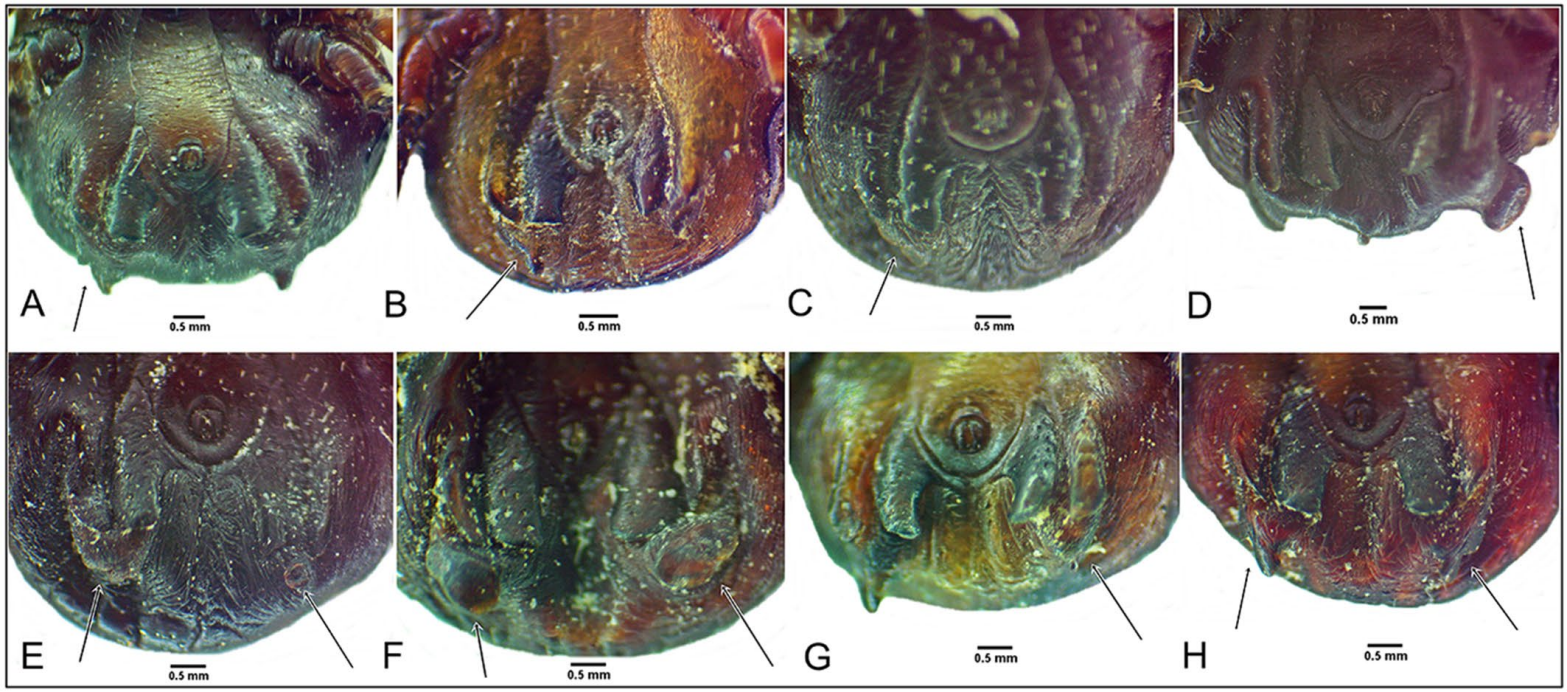
Subanal plate anomalies in *Hyalomma dromedarii* male, ventral view (**A**–**H**); adanal plate anomaly in *H. dromedarii* male, ventral view (**B**, **E**); sclerotized formation in ventral plate and anal groove anomaly on *H. dromedarii* male, ventral view (**D**)

**Fig. 4:**
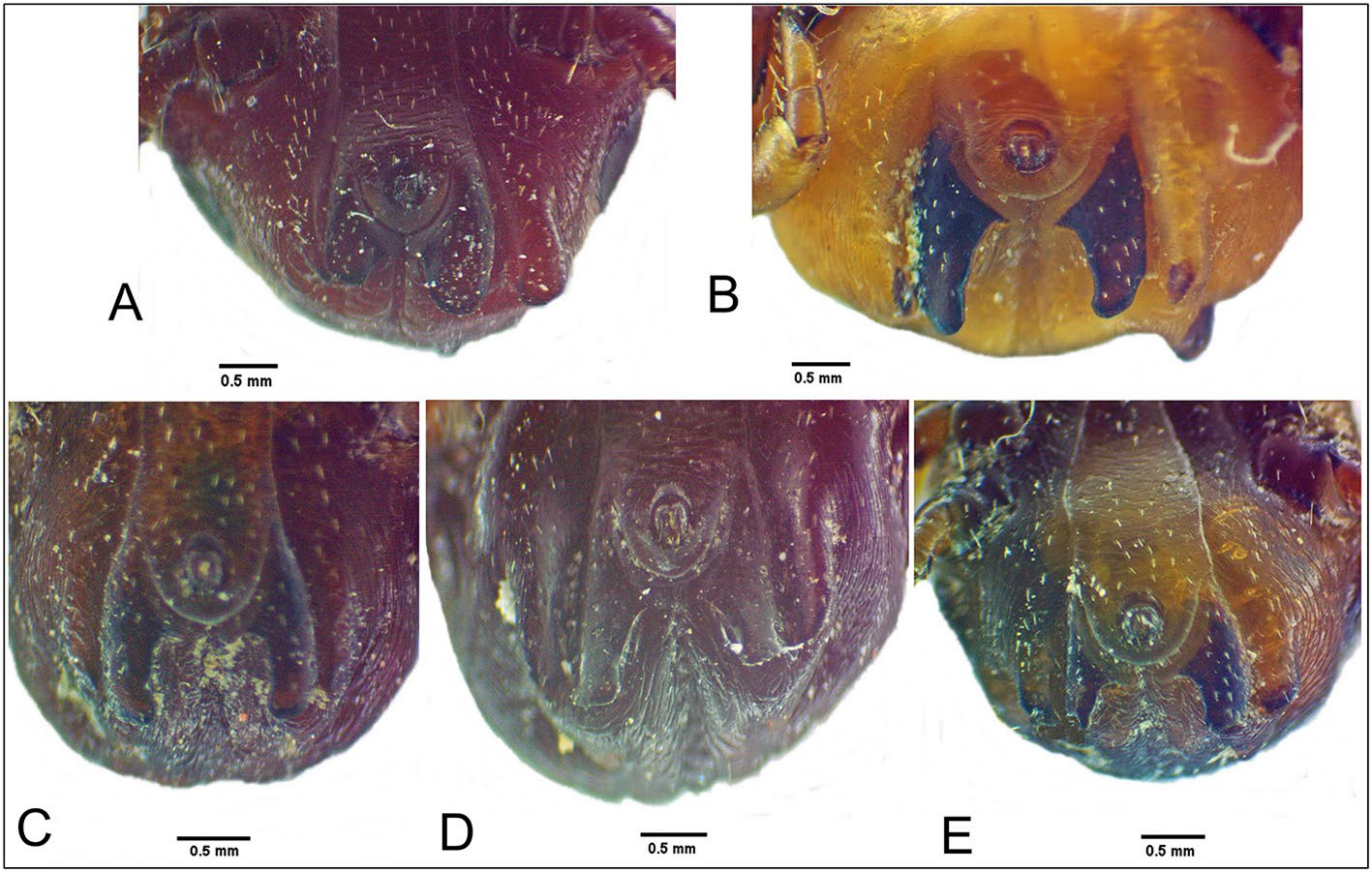
Missing subanal and accessory adanal plates in *Hyalomma rufipes* male, ventral view (**A**); missing subanal plates in *H. dromedarii* male, ventral view (**B**, **C**); adanal plate anomalies in *H. dromedarii* male, ventral view (**D**, **E**)

Subanal plate anomalies were the most common local abnormality observed and were most prevalent in *H. dromedarii* males (n = 20, 38.5%) (Figs. 2 and 3). Fusion of adanal and accessory adanal plate was noted in one *H. dromedarii* male on the right side (Fig. 2A). Sclerotized formation of the ventral plate and an anomaly in anal groove form were both noted in one *H. dromedarii* male (Fig. 3D). Adanal plate anomalies were seen in 0.4% of the ticks, including four *H. dromedarii* males and one *H. rufipes* male (Figs. 3B and E and 4A, D, E). One *H. rufipes* male exhibited a missing right accessory adanal plate (Fig. 4A). Absent subanal plates were noted in two *H. dromedarii* males and one *H. rufipes* male (Fig. 4A–C).

**Fig. 5:**
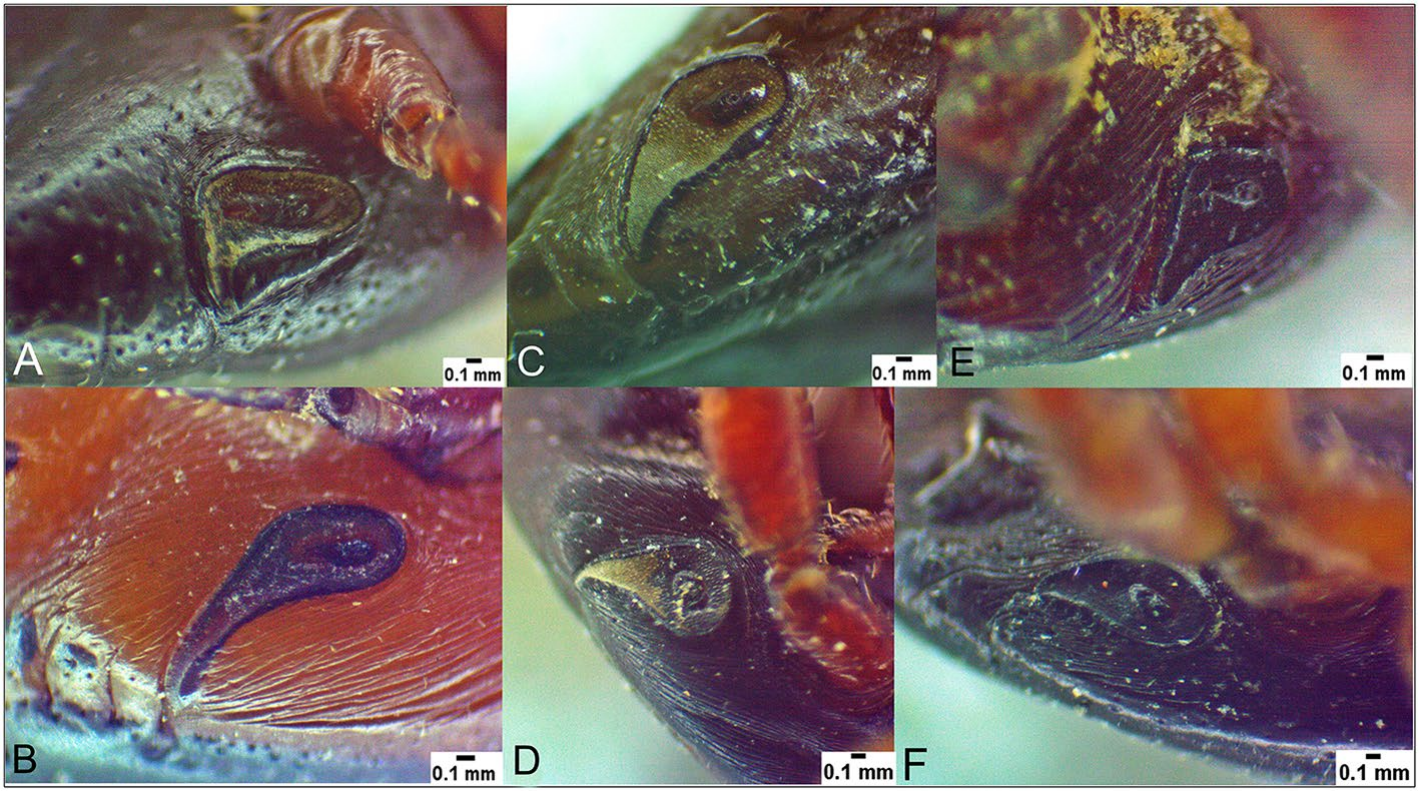
Spiracles of *Hyalomma dromedarii*, normal female (**A**); normal male (**B**); abnormal female (**C**); abnormal males (**D**–**F**)

Spiracle anomalies were observed in one female (Fig. 5C) and three males *H. dromedarii* (Fig. 5D–F). In the female specimen, the right spiracle showed unusual shape with dorsal prolongation sharply pointed and longer than normal (Fig. 5A, C). Two males had unusual shape of the left spiracle, which had dorsal prolongation slightly shorter than normal (Fig. 5D, E), and one male had the right spiracle with dorsal prolongation sharply pointed and shorter than normal (Fig. 5F). The ratios of spiracular plate length to dorsal prolongation in the three abnormal males were 1.3:1 (Fig. 5D), 1.6:1 (Fig. 5E), and 3:1 (Fig. 5F), whereas the ratio in the normal male was 1:1.5 (Fig. 5B).

**Fig. 6:**
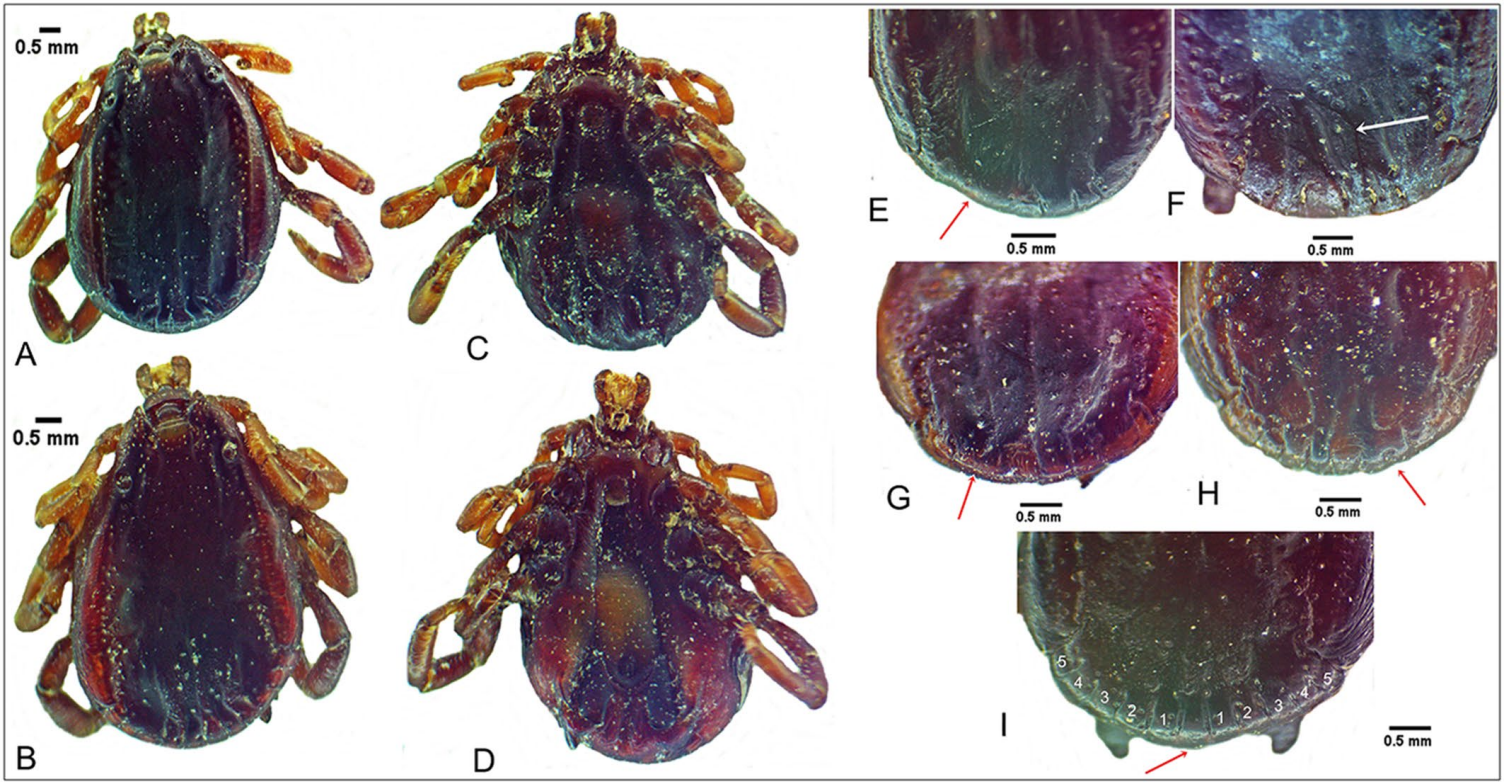
Asymmetries in *Hyalomma dromedarii* male, dorsal view (**A**, **B**), ventral view (**C**, **D**); festoon anomalies in *H. dromedarii* male, dorsal view (**E**, **G**–**I**); parma anomalies in *H. dromedarii* male, dorsal view (**E**, **G**, **I**); postermedian groove anomalies in *H. dromedarii* male, dorsal view (**F**, **I**)

Fusion of festoons was observed in three *H. dromedarii* males (Fig. 6E, G, H), whereas one *H. dromedarii* male exhibited 10 festoons around the parma (Fig. 6I). Furthermore, parma anomalies were noted in three *H. dromedarii* males (Fig. 6E, G, I). One *H. dromedarii* male exhibited an abnormal shape of postermedian groove and exoskeleton anomaly (Fig. 6F), and another *H. dromedarii* male showed postermedian groove not reaching parma (Fig. 6I).

## General anomalies

Asymmetry was observed in 3.8% (n = 2) of *H. dromedarii*. The *H. dromedarii* males showed slight asymmetry on the right side (Fig. 6A–D), whereas in one (Fig. 6B, D) asymmetry was also associated with local anomaly (subanal plate anomalies) (Fig. 3H).

**Fig. 7:**
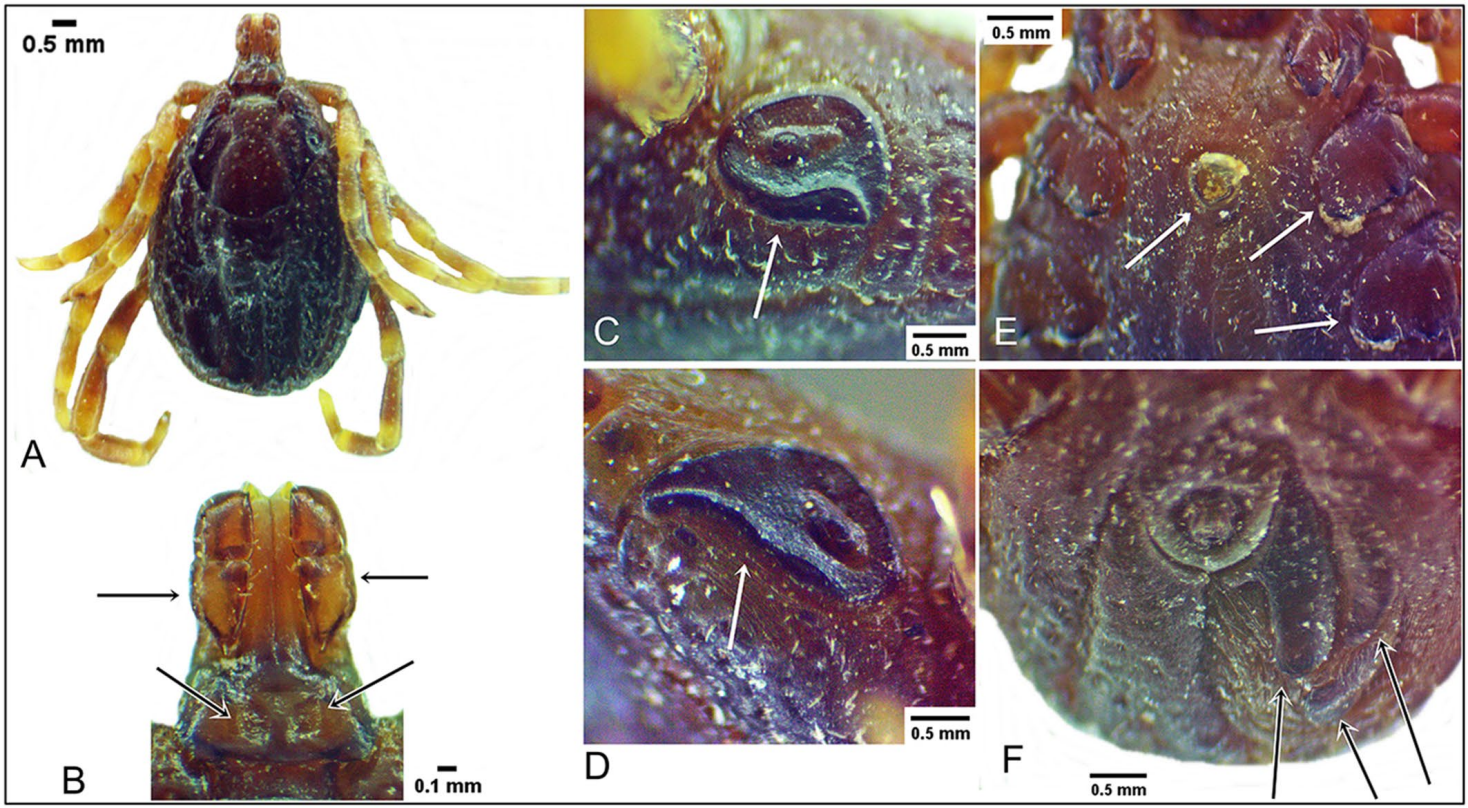
Gynandromorphism in *Hyalomma dromedarii*, dorsal view (**A**); capitulum dorsal view (**B**); normal shape of female right spiracle (**C**); abnormal shape of left spiracle for both sexes (**D**); abnormal shape of the genital aperture and coxae with female features (**E**); the male characters on the left side with the presence of the ventral plates, ventral view (**F**)

**Fig. 8:**
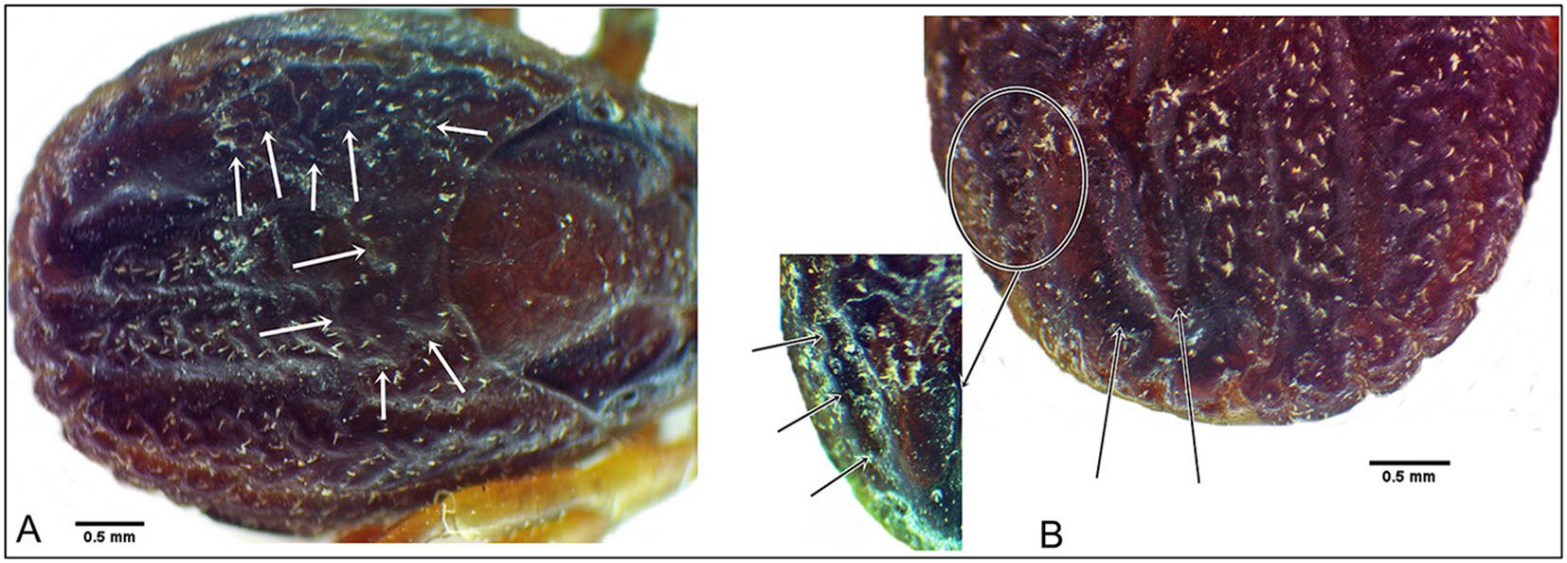
Gynandromorphic *Hyalomma dromedarii* with the pieces of male scutum (marked with white arrows) embedded in female alloscutum, dorsal view (**A**); the male characters on the left side with the presence of a posterior ridge, paramedian groove, and lateral groove, dorsal view (**B**)

Gynandromorphism was observed in one specimen of *H. dromedarii* (Figs. 7 and 8). This gynandromorphic specimen had the size of a female (8.4 mm long), and the ratio of scutum length-to-width is 0.82:1 (Fig. 7A), whereas the length of male specimens usually did not exceed 8.3 mm. Capitulum on dorsal view showed the features of a female, with oval porose areas in basis capitulum and the three segments of palps (segment II longer than segments I and III, also the apex of segment III broadly rounded) displayed female features (Fig. 7B). The right spiracle displayed female features (Fig. 7C), whereas the left one had an abnormal shape for both sexes (Fig. 7D). The genital aperture had abnormal shape for both sexes; however, the coxae showed the features of a female (Fig. 7E). Ventral male features were observed in ventral plates: one adanal, one subanal, and one accessory adanal plates were present on the left side only (Fig. 7F). Female features were generally present dorsally, but with pieces of male conscutum embedded in the female alloscutum (Fig. 8A). The specimen displayed male features on the dorsal left side, with the presence of a posterior ridge, well-defined paramedian groove, and short lateral groove. Moreover, left dorsal festoons were of a typical shape for males (Fig. 8B). The number of female characters in the gynandromorphic specimen was more than eight, whereas the male characters were only three. So, the gynandromorphic specimen looks like a female at first view, due to the dominance of female morphological characters.

## Discussion

In this study, we report morphological abnormalities in 4.2% of total ticks collected from dromedary camels in Aswan, Egypt. Our findings are in agreement with previous studies (Labruna et al. 2000; Alekseev et al. 2007; Keskin et al. 2016; Chitimia-Dobler et al. 2017; Azzi et al. 2019; Salceda-Sanchez et al. 2020), which indicated morphological anomalies occur in a range of 1 to 48% among ixodid tick populations. However, these results are not in accord with other studies that reported morphological anomalies in 0.03–0.62% of ixodid tick populations (Latif et al. 1988; Guglielmone et al. 1999; Dergousoff and Chilton 2007; Nowak-Chmura 2012; Kar et al. 2015; Chitimia-Dobler and Pfeffer 2017; Munoz-Leal et al. 2018; Balinandi et al. 2019; Molaei and Little 2020; Shuaib et al. 2020; Laatamna et al. 2021). The anomaly prevalence is different from study to study according to the tick species and number of investigated ticks. Ticks with anomalies are found during studies, which do not have tick anomalies in focus, therefore, it is important to be observed and described. Although several tick surveillance programs have been implemented in various ecological zones throughout the country (Okely et al. 2022), this is the first report on morphological abnormalities in ticks from Egypt.

Leg atrophy was reported in several genera of hard ticks such as *Dermacentor*, *Ixodes*, *Rhipicephalus*, *Amblyomma*, and *Hyalomma* (Nowak-Chmura 2012; Kar et al. 2015; Chitimia-Dobler et al. 2017; Chitimia-Dobler and Pfeffer 2017). Previous studies have documented atrophy of legs in *Hyalomma* species such as *H. impeltatum* (Shuaib et al. 2020), *H. marginatum* (Kar et al. 2015; Keskin et al. 2016), and *H. scupense* (Kar et al. 2015); however, no previous cases of such abnormalities have been documented in *H. dromedarii* and *H. rufipes*.

Ectromely is described in a *H. dromedarii* male during this study. This anomaly has been recorded previously in *H. marginatum* in nature (Buczek et al. 1991) and was also observed in *H. marginatum* larvae experimentally exposed to 90% RH and 25 °C, kept under these conditions during the whole embryonic development (Buczek 2000). Ectromely was also detected in other species such as *Amblyomma cajennense*, *Amblyomma neumanni*, *Amblyomma americanum*, *A. lepidum*, *Dermacentor andersoni*, *Ixodes scapularis*, *Rhipicephalus decoloratus*, *Rhipicephalus sanguineus*, *Rhipicephalus microplus*, and *Rhipicephalus evertsi evertsi* (Guglielmone et al. 1999; Dergousoff and Chilton 2007; Balinandi et al. 2019; Molaei et al. 2020; Shuaib et al. 2020). This study describes for the first time ectromely in a *H. dromedarii* male.

Ventral plate anomalies among *Hyalomma* species were documented in *H. dromedarii* and *H. impeltatum* as missing subanal plates and atrophy of adanal and accessory adanal plates (Shuaib et al. 2020). Notably, we observed one *H. rufipes* male with missing accessory adanal plate and subanal plate. Also, the same specimen displayed anomaly in adanal plate form. To our knowledge, this is the first report of ventral plate anomalies in *H. rufipes*. In addition, several other anomalies were observed in *H. dromedarii* males including fusion of adanal and accessory adanal plates on one side of the body, missing subanal plates, and anomalies in adanal and subanal plate forms. Ventral plate anomalies were detected for the first time in male *H. dromedarii* specimens from Egypt during this study.

Anal groove anomalies were documented in an *Amblyomma latum* male collected from exotic reptiles in Poland (Nowak-Chmura 2012) and *Ixodes ricinus* females and nymphs collected by the flagging method in Germany (Chitimia-Dobler et al. 2017), but no previous studies have reported such anomalies in *Hyalomma* species. Sclerotized formation in the ventral plate was observed in one *H. dromedarii* male in our study and in the same species reported earlier from Sudan (Shuaib et al. 2020). In addition, anomalies of the dorsal prolongation of spiracles were seen in one female and three *H. dromedarii* males. Various types of anomalies of spiracles have been reported, such as atrophy or lack of one spiracle in *H. aegyptium* and *H. excavatum* (Keskin et al. 2016). Spiracular abnormalities have also been described in *Amblyomma hebraeum*, *Amblyomma longirostre*, *H. scupense*, *H. marginatum*, and *Rhipicephalus turanicus* (Campana-Rouget 1959b; Kar et al. 2015).

Fusion of festoons was detected in three specimens of *H. dromedarii*, but there is no such previous report for this species. However, this case was observed in *H. marginatum* (Keskin et al. 2016). Postermedian groove and parma anomalies are reported during this study in *Hyalomma* species.

Asymmetries of the idiosoma were observed in several ixodid ticks such as *Amblyomma parvum*, *Amblyomma tigrinum*, *A. cajennense*, *A. neumanni*, *A. latum*, *A*. *lepidum*, *Haemaphysalis parva*, *H. marginatum*, *H. scupense*, *H. excavatum*, *I. ricinus*, *Rhipicephalus bursa*, *R. decoloratus*, and *R. evertsi evertsi* (Buczek et al. 1991; Guglielmone et al. 1999; Nowak-Chmura 2012; Kar et al. 2015; Keskin et al. 2016; Chitimia-Dobler et al. 2017; Shuaib et al. 2020). These cases occur in nature due to many factors, including – but not limited to – missing tissues, underdevelopment of structures and organs in the idiosoma, or abnormal development during metamorphosis from the immature stages to the adult (Kar et al. 2015). Additionally, these factors may result in the formation of other types of abnormalities beyond idiosomal asymmetries. Two cases of asymmetry in the idiosoma are detected in *H. dromedarii* males in Egypt during this study.

Gynandromorphism has been documented in approximately 80 field-collected specimens of ixodid ticks (Salceda-Sánchez et al. 2020), and the frequency of gynandromorphism in *Amblyomma* and *Hyalomma* species is relatively higher (Labruna et al. 2002; Keskin et al. 2012; Muñoz-Leal et al. 2018). No previous reports of gynandromorphism were made in *H. dromedarii* collected from a dromedary camel. According to the classification system for tick gynandromorphs reported by Campana-Rouget (1959a), the gynandromorphic specimen of this study is considered a deuterogynander intrigue, where characters of male parts are reduced to a quadrant. Several cases of gynandromorphism in *Hyalomma* species have been described in *H. marginatum* (Keskin et al. 2012, 2016; Buczek et al. 2014; Kar et al. 2015), *Hyalomma asiaticum* (Chen et al. 2015), *Hyalomma truncatum* (Kostrzewski et al. 1986; Clarke and Rechav 1993), *Hyalomma anatolicum* (Kumar and Nagar 1979), and *Hyalomma savignyi* (Feldman-Muhsam 1950). However, the deuterogynander type has only been previously documented in *H. truncatum* infesting rabbits in the laboratory (Clarke and Rechav 1993). The deuterogynander gynandromorphic type has also been described for *Rhipicephalus sanguineus* sensu lato and *Rhipicephalus simus* (= *praetextatus*) (Campana-Rouget 1959a, b; Salceda-Sánchez et al. 2020; Ortíz-Giraldo et al. 2022). This study documents gynandromorphism in ixodid ticks from Egypt.

These anomalies might be due to climatic conditions like high temperature and dry weather, as Aswan is one of the hottest and driest cities in the whole world (https://weatherspark.com/). It has a hot dry desert, and it suffers from thermal stress throughout the whole year (El Menshawy et al. 2022). Climate change influences could probably increase the prevalence of morphological anomalies in structures of ticks (Nowak-Chmura 2012; Kar et al. 2015; Shuaib et al. 2020), so studies of the environmental factors under changing climate that may have led to these phenomena in Egypt are important.

In conclusion, the present study reports several types of morphological abnormalities in *H. dromedarii* and *H. rufipes* collected from dromedary camels in Egypt. It is recommended to intensify field surveillance programs for collecting tick specimens from different ecological zones in Egypt and monitoring anomalies in ticks from different hosts and zones.

## References

[CR1] Alekseev AN, Dubinina HV, Jaaskelainen AE, Vapalahti O, Vaheri A (2007). First report on tick-borne pathogens and exoskeletal anomalies in *Ixodes persulcatus* Schulze (Acari: Ixodidae) collected in Kokkola Coastal region, Finland. Int J Acarol.

[CR2] Apanaskevich DA, Horak IG (2008). The genus *Hyalomma* Koch, 1844: V. Re-evaluation of the taxonomic rank of taxa comprising the *H.* (*Euhyalomma*) *marginatum* Koch complex of species (Acari: Ixodidae) with redescription of all parasitic stages and notes on biology. Int J Acarol.

[CR3] Apanaskevich DA, Schuster AL, Horak IG (2008). The genus *Hyalomma*: VII. Redescription of all parasitic stages of *H.* (*Euhyalomma*) *dromedarii* and *H. (E) schulzei* (Acari: Ixodidae). J Med Entomol.

[CR4] Azzi CFG, Aprigio CJL, Souza RVD, Borsoi ABP, Garcia KB, Ferreira A, Amorim M, Oliveira SVD, Gazeta GS (2019). Morphological abnormality in larvae of *Amblyomma oblongoguttatum* (Acari: Ixodidae). Vet Not.

[CR5] Balinandi S, Mugisha L, Johnson B, William K, Teddy N, Bakkes DK, Lutwama JJ, Chitimia–Dobler L, Malmberg M (2019). General and local morphological anomalies in *Amblyomma lepidum* (Acari: Ixodidae) and *Rhipicephalus decoloratus* infesting cattle in Uganda. J Med Entomol.

[CR6] Buczek A (2000). Experimental teratogeny in the tick *Hyalomma marginatum marginatum* (Acari: Ixodida: Ixodidae) effect of high humidity on embryonic development. J Med Entomol.

[CR10] Buczek A, Siuda K, Alsied S (1991). Morphological anomalies in ticks (Acari: Ixodida) collected from natural environment. Wiad Parazytol.

[CR9] Buczek A, Bartosik K, Kuczyński P (2013). Evaluation of the effect of various concentrations of selected pyrethroids on the development of *Dermacentor reticulatus* eggs and larvae. Ann Agric Environ Med.

[CR8] Buczek A, Bartosik K, Buczek S (2014). Four gynandromorphs of *Hyalomma marginatum marginatum* ticks (Acari: Ixodidae) from a laboratory colony. J Nat Hist.

[CR7] Buczek A, Bartosik K, Buczek AM, Buczek W, Kulina D (2019). Abnormal development of *Hyalomma marginatum* ticks (Acari: Ixodidae) induced by plant cytotoxic substances. Toxins.

[CR11] Campana-Rouget Y (1959). Teratology of ticks. Ann Parasitol Hum Comp.

[CR12] Campana-Rouget Y (1959). Teratology of ticks. Ann Parasitol Hum Comp.

[CR13] Chen Z, Li YQ, Ren QY, Luo J, Hu Y, Li K, Liu GY, Luo JX, Liu J, Yin H (2015). Morphological characteristics of normal and gynandromorphic *Hyalomma asiaticum* Schulze and Schlottke, 1930. Korean J Parasitol.

[CR15] Chitimia-Dobler L, Pfeffer M (2017). Gynandromorphism and local morphological abnormalities in *Dermacentor reticulatus* (Acari: Ixodidae). Syst Appl Acarol.

[CR14] Chitimia-Dobler L, Bestehorn M, Broker M, Borde J, Molcanyi T, Andersen NS, Pfeffer M, Dobler G (2017). Morphological anomalies in *Ixodes ricinus* and *Ixodes inopinatus* collected from tick-borne encephalitis natural foci in Central Europe. Exp Appl Acarol.

[CR16] Chong ST, Kim HC, Suh SJ, Klein TA, Robbins RG (2020). Morphological abnormalities in ticks (Acari: Ixodidae) from the Republic of Korea. Syst Appl Acarol.

[CR17] Clarke FC, Rechav Y (1993). Gynandromorphism in *Hyalomma truncatum* (Acarl: Ixodidae). Int J Trop Insect Sci.

[CR18] Dergousoff SJ, Chilton NB (2007). Abnormal morphology of an adult Rocky Mountain wood tick, *Dermacentor andersoni* (Acari: Ixodidae). J Parasitol.

[CR19] Diyes CP, Rajakaruna RS (2021). Teratological anomalies of an adult Asiatic blue tick, *Rhipicephalus microplus* (Acari: Ixodidae). Syst Appl Acarol.

[CR20] El Menshawy AS, Mohamed AF, Fathy NM (2022). A comparative study on green wall construction systems, case study: South valley campus of AASTMT. Case Stud Constr Mater.

[CR21] Feldman-Muhsam B (1950). On some abnormalities in *Hyalomma savignyi*. Parasitology.

[CR22] Gothe R (1967). Ticks in the South African Zoological Survey Collection: Part XIII. Gynanders of *Boophilus decoloratus* (Koch, 1844) and *Amblyomma hebraeum* Koch, 1844. Onderstepoort J Vet Res.

[CR23] Guglielmone AA, Castella J, Mangold AJ, Estrada–Pena A, Vinabal AE (1999). Phenotypic anomalies in a collection of neotropical ticks (Ixodidae). Acarologia.

[CR24] Hoogstraal H (1956) African ixodoidea, vol 1. Department of the Navy, Bureau of Medicine and Surgery

[CR25] Kar S, Akyildiz G, Yilmazer N, Shaibi T, Gargili A, Vatansever Z (2015). External morphological anomalies in ixodid ticks from Thrace, Turkey. Exp Appl Acarol.

[CR26] Keskin A, Bursali A, Tekin S (2012). A case of gynandromorphism in *Hyalomma marginatum* Koch, 1844 (Acari: Ixodidae). J Parasitol.

[CR27] Keskin A, Simsek E, Bursali A, Keskin A (2016). Morphological abnormalities in ticks (Acari: Ixodidae) feeding on humans in Central Black Sea region, Turkey. Zoomorphology.

[CR28] Kittelmann S, Buffry AD, Franke FA, Almudi I, Yoth M, Sabaris G, Couso JP, Nunes MD, Frankel N, Gómez-Skarmeta JL, Pueyo-Marques J (2018). Gene regulatory network architecture in different developmental contexts influences the genetic basis of morphological evolution. PLoS Genet.

[CR29] Kostrzewski MW, Van Niekerk JP, Rechav Y (1986). A case of gynandromorphism in *Hyalomma truncatum* (Acari: Ixodidae). J Med Entomol.

[CR30] Kumar K, Nagar SK (1979). Two kinds of Gynandromorphs in ticks *Boophilus microplus* (Canestrini, 1888) and *Hyalomma anatolicum* Koch, 1844. Acarologia.

[CR31] Laatamna A, Bakkes DK, Chitimia-Dobler L (2021). Morphological anomalies in *Rhipicephalus sanguineus* s.s. (Acari: Ixodidae) collected from dogs in steppe and high plateaus regions, Algeria. Exp Appl Acarol.

[CR32] Labruna MB, Homem VSF, Heinemann MB, Ferreira Neto JS (2000). A case of gynandromorphism in *Amblyomma oblongoguttatum* (Acari: Ixodidae). J Med Entomol.

[CR33] Labruna MB, Ribeiro AF, Cruz MV, Camargo LMA, Camargo EP (2002). Gynandromorphism in *Amblyomma cajennense* and *Rhipicephalus sanguineus* (Acari: Ixodidae). J Parasitol.

[CR34] Larson SR, Paskewitz SM (2016). Teratological nymphal *Ixodes scapularis* (Acari: Ixodidae) from Wisconsin. J Med Entomol.

[CR35] Latif AA, Dhadialla TS, Newson RM (1988). Abnormal development of *Amblyomma variegatum* (Acarina: Ixodidae). J Med Entomol.

[CR36] Martini A, Baldassari N, Baronio P (1999). Gynandromorphism and its manifestations in Diprionid (Hymenoptera). Boll Ist Ent G Grandi Univ Bologna.

[CR37] Molaei G, Little EAH (2018). A nine-legged tick: report of a morphological anomaly in the blacklegged tick, *Ixodes scapularis* (Acari: Ixodidae) from the northeastern United States. Ticks Tick Borne Dis.

[CR38] Molaei G, Little EAH (2020). A case of morphological anomalies in *Amblyomma americanum* (Acari: Ixodidae) collected from nature. Exp Appl Acarol.

[CR39] Molaei G, Little EAH, Staford KC, Gaff H (2020). A seven-legged tick: report of a morphological anomaly in *Ixodes scapularis* (Acari: Ixodidae) biting a human host from the Northeastern United States. Ticks Tick Borne Dis.

[CR40] Muñoz-Leal S, Martins TF, Luna LR, Rodriguez A, Labruna MB (2018). A new collection of *Amblyomma parvitarsum* (Acari: Ixodidae) in Peru, with description of a gynandromorph and report of *Rickettsia* detection. J Med Entomol.

[CR41] Neumann LG (1899). Anomalie d’ixodides. Arch Parasitol.

[CR42] Nowak-Chmura M (2012). Teratological changes in tick morphology in ticks feeding on exotic reptiles. J Nat Hist.

[CR43] Okely M, Anan R, Gad-Allah S, Samy AM (2021). Hard ticks (Acari: Ixodidae) infesting domestic animals in Egypt: diagnostic characters and a taxonomic key to the collected species. Med Vet Entomol.

[CR44] Okely M, Chen Z, Anan R, Gad-Allah S (2022). Updated checklist of the hard ticks (Acari: Ixodidae) of Egypt, with notes of livestock host and tick-borne pathogens. Syst Appl Acarol.

[CR45] Oliver JH, Delfin ED (1967). Gynandromorphism in *Dermacentor occidentalis* (Acari: Ixodidae). Ann Entomol Soc Am.

[CR46] Ortíz-Giraldo M, Cardona-Giraldo A, Ez-Guarín DV, Ramírez-Chaves HE, Rivera-Páez FA (2022). A gynandromorph of the brown dog tick, *Rhipicephalus sanguineus* s.l. (Latreille, 1806) from Colombia. Syst Appl Acarol.

[CR47] Ren Q, Chen Z, Luo J, Liu G, Guan G, Yin H, Luo J (2016). Abnormal development of *Haemaphysalis qinghaiensis* (Acari: Ixodidae). J Insect Sci.

[CR48] Salceda-Sanchez B, Sanchez-Montes S, Soto-Gutierrez JJ, Sandoval-Espinosa MR (2020). A case of gynandromorphism in *Rhipicephalus sanguineus* s.l. from Mexico. Exp Appl Acarol.

[CR49] Shuaib YA, Isaa MH, Ezz–Eldin MIE, Abdalla MA, Bakhiet AO, Chitimia-Dobler L (2020). Morphological abnormalities in ticks (Acari: Ixodidae) collected from domestic animal species in Sudan. Exp Appl Acarol.

[CR50] Walker AR, Bouattour A, Camicas JL, Estrada-Pena A, Horak IG, Latif AA, Pegram RG, Preston PM (2003) Ticks of domestic animals in Africa: guide to identification of species. ICTTD

[CR51] Wang X, Chen Z, Liu J (2019). A record of morphological anomalies in the tick *Dermacentor nuttalli* Olenev (Acari: Ixodidae). Preprints.

[CR52] Weather Spark https://weatherspark.com/y/97255/Average-Weather-in-Aswan-Egypt-Year-Round

[CR53] Zharkov SD, Dubinina HV, Alekseev AN, Jensen PM (2000). Anthropogenic pressure and changes in *Ixodes* tick populations in the Baltic region of Russia and Denmark. Acarina.

